# Azocasein Substrate for Determination of Proteolytic Activity: Reexamining a Traditional Method Using Bromelain Samples

**DOI:** 10.1155/2016/8409183

**Published:** 2016-01-27

**Authors:** Diego F. Coêlho, Thais Peron Saturnino, Fernanda Freitas Fernandes, Priscila Gava Mazzola, Edgar Silveira, Elias Basile Tambourgi

**Affiliations:** ^1^Chemical Engineering School, Campinas State University (UNICAMP), Avenida Albert Einstein 500, 13083-852 Campinas, SP, Brazil; ^2^Faculty of Pharmaceutical Sciences, Campinas State University (UNICAMP), Rua Sérgio Buarque de Holanda 250, 13083-859 Campinas, SP, Brazil; ^3^Biochemistry and Genetics Institute, Federal University of Uberlândia (UFU), Avenida Getúlio Vargas, 230 Centro, 38700-128 Patos de Minas, MG, Brazil

## Abstract

Given the importance of protease's worldwide market, the determination of optimum conditions and the development of a standard protocol are critical during selection of a reliable method to determine its bioactivity. This paper uses quality control theory to validate a modified version of a method proposed by Charney and Tomarelli in 1947. The results obtained showed that using azocasein substrate bromelain had its optimum at 45°C and pH 9 (Glycine-NaOH 100 mM). We also quantified the limit of detection (LoD) and limit of quantification (LoQ) in the above-mentioned optimum (0.072 and 0.494 mg·mL^−1^ of azocasein, resp.) and a calibration curve that correlates optical density with the amount of substrate digested. In all analysed samples, we observed a significant decrease in response after storage (around 17%), which suggests its use must be immediately after preparation. Thus, the protocol presented in this paper offers a significant improvement, given that subjective definitions are commonly used in the literature and this simple mathematical approach makes it clear and concise.

## 1. Introduction

Because proteases represent the largest and most important segment in the industrial enzyme market [1], the consolidation of a reliable method to evaluate its quality is obviously of extreme importance. These enzymes are used in detergents, food processing, and leather industry, as biocatalysts in organic synthesis, and, among many other applications, as therapeutics because their roles are involved in key decisions throughout an organism in several physiological and metabolic processes [2].

The global market for industrial enzymes is expected to reach US $7.1 billion by 2018 [3] and is traditionally divided into three segments: food, technical, and feed enzymes. In 2000, technical enzymes used in detergent, leather, textile, and personal care industries accounted for 65% [4] of the total sales (approximately US $1.5 billion [5]), while food enzymes, which include enzymes used in dairy, brewing, wine, and juices, were valued at 25% and feed enzymes (used in animal feeds) contributed with 10%.

Nearly 70 years ago, Charney and Tomarelli [6] proposed the use of an azoprotein (a protein coupled with diazotized aryl amines) for the determination of proteolytic activity. The digestion of a solution with such proteins releases the chromophoric group, which is soluble in trichloroacetic acid and gives it a red-orange colour.

The method itself relies on the reaction between the substrate and an enzyme under its optimum temperature/pH for a given time. The solution colour intensity, read at 440 nm, is a function of the amount of azoprotein digested, since all proteins remaining precipitate after the addition of trichloroacetic acid.

The method is still one of the most reliable methods to study the proteolytic activity of enzymes [7, 8] due its colour stability and no need of chromogenic reagents. Besides, the sulphanilamide-azocasein's preparation is no longer necessary, since it is now available widely in the market.

However, the available protocols that describe thoroughly the method still are lacking in presenting the evaluation of its analytical parameters, required for method validation. Thus, this study aims to review and validate the azocasein method to establish its detection and quantification limits, in addition to reagent storage stability and a quantitative definition of enzymatic activity.

## 2. Materials and Methods

### 2.1. Bromelain Sample and Other Chemicals

Bromelain (catalogue B5144) and azocasein (catalogue A2765) obtained from Sigma-Aldrich (St. Louis, USA) were chosen as standards for these studies, being used to prepare stock solutions at different pH. Unless specified, all other reagents were also obtained from Sigma-Aldrich.

### 2.2. Substrate Solution

Given the nature of this study, the amount of powdered substrate and buffer used will depend on the concentration and pH of each experiment. The substrate's pH and concentration are part of the studied variables and are described in the following methods. All pH buffers were prepared following common protocols described elsewhere [9].

Basically, 4 mL of ethanol is added to the powdered substrate in a 120 mL beaker and is stirred using a magnetic stirrer to solubilise all aggregated protein and is then diluted with 96 mL of appropriated buffer (100 mM).

### 2.3. Bromelain Stock Solution

Bromelain stock solution was prepared following a modified version of a method described by Hale et al. [10]. The 1 mg·mL^−1^ enzyme solution was prepared using a 100 mM buffer of different pH (since it was also under investigation). Concentration was chosen based on its maximum solubility at experimental conditions.

### 2.4. Enzymatic Assay

The method consists in mixing equal volumes of substrate and enzymatic sample at a given temperature and pH that corresponds to the optimum conditions of the enzyme under investigation. For practical reasons we chose 125 *μ*L, as it is small enough to avoid wasting resources and does not compromise the method's precision.

The kinetics of the digestion were studied during 420 minutes using substrate's concentration in a range from 0.1 to 3.0% (w/w) in order to determine a suitable time of digestion.

The reaction was terminated adding 750 *μ*L of 5% trichloroacetic acid (TCA) to the enzyme-substrate mixture. The coagulated protein was removed by centrifugation at 2000 ×g for 10 min at room temperature. The obtained supernatant was then added to a 0.5 N NaOH solution using a 1 : 1 (v/v) ratio and its absorbance was read at 440 nm.

The blank was obtained by mixing the TCA to the substrate prior to the enzyme addition.

### 2.5. Optimum pH and Temperature for Bromelain

The optimum pH and temperature for assaying bromelain's activity were determined by performing a full factorial design of experiments using both variables in two levels and three central points. The pH ranged from 6 to 8 and temperature from 25°C to 45°C in the factorial design. Temperature was kept constant during substrate digestion by using a Techne^*®*^ Dri-Block^*®*^ heater, model DB-3D.

This design was extended to a central composite design, which had its variable's range adjusted based on the results of the first design. All statistical data was generated and analysed using R [11], coupled with R-Studio [12], and using packages akima [13], DoE.base [14], ggplot2 [15], and RColorBrewer [16].

### 2.6. Calibration Curve

Using the curves of azocasein digestion obtained previously (as described in the topic Enzymatic Assay), a correlation between the colour intensity and the substrate concentration was created.

The principle is simple: if the enzymes digest the substrate for enough time, we would achieve the solution maximum colour intensity, since all chromophoric groups had their bonds to the protein broken and thus are soluble in TCA. This satisfies the assumption made in azocasein's original protocol [6], which states that a completely digested azocasein solution has the same colour intensity as an undigested sample.

The calibration curve is obtained by plotting the optical density measured when the time of digestion was 420 min and the concentration of substrate at *t* = 0.

### 2.7. Detection and Quantification Limits

The limit of detection (LoD) and limit of quantification (LoQ) for the protocol were based on the standard deviation of the response and the slope of the mean of calibration curves, following ICH^*∗*^'s guidelines [17], and are given by the equations below:

(1) LoD=3.3·σs,LoQ=10·σs,

where *σ* is the standard deviation of the response and *s* is the slope of the calibration curve. As described by ICH, the residual standard deviation of a regression line can be used as the standard deviation during calculations.

### 2.8. Stability Assays

Stability assays followed the protocols described in a document provided by the US Department of Health and Human Services called Guidance for Industry: Bioanalytical Method Validation [18].

*Short-Term Temperature Stability*. Three aliquots of each of the low and high concentrations were thawed at room temperature, kept for 8 hours, and then analysed.

*Long-Term Stability*. The storage time in a long-term stability was evaluated within an interval of six weeks, time usually necessary to perform a whole batch of our routine experiments. Long-term stability was determined by storing three aliquots of each of the low and high concentrations at 5°C. To avoid contamination, each sample was stored in its own vial and analysed on six separate occasions.

*Freeze and Thaw Stability*. Three aliquots at each of the low and high concentrations were stored at −20°C for 24 hours and thawed unassisted at room temperature. When completely thawed, the samples were refrozen for 24 hours under the same conditions. The freeze-thaw cycle was repeated two more times and then analysed on the third cycle.

## 3. Results and Discussion

### 3.1. Optimum Conditions

The study and determination of bromelain's biochemical properties have been studied extensively before through several methods but our interest was to determine the optimum conditions specifically for the substrate under investigation to evaluate it at its best.

**Figure 1:**
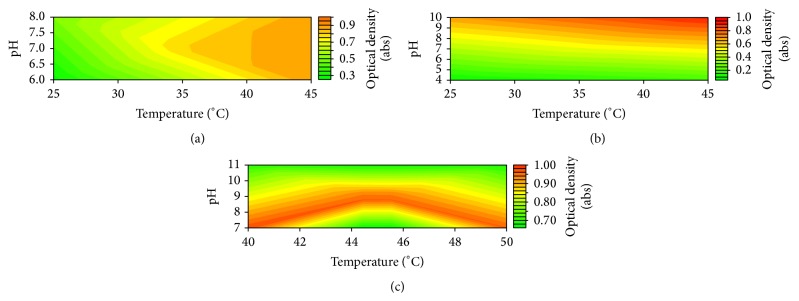
Response contour of conditions optimisation for bromelain solution.

Figure 1(a) corresponds to results obtained from the first experimental design and shows that at such variable's range the pH seems to have no influence over the enzyme activity.

Then we modified the experimental design by increasing the pH's range in order to confirm the observation. However, the enzyme showed some increase in its activity at basic pH (Figure 1(b)) and served to establish the variables range for the central composite design (CCD) shown in Table 1.

Figure 1(c) shows clearly that bromelain has an impressively wide range of pH and temperature that can digest azocasein substrate with no apparent loss in its sensitivity. It also shows that bromelain is still active at moderately high temperatures [19]. Due to local operational reasons we chose pH 9 and 45°C as the conditions to be used in the next steps of this study. For this case, pH 9 Glycine-NaOH (100 mM) buffer was used during substrate preparation.

### 3.2. Calibration Curve

**Figure 2:**
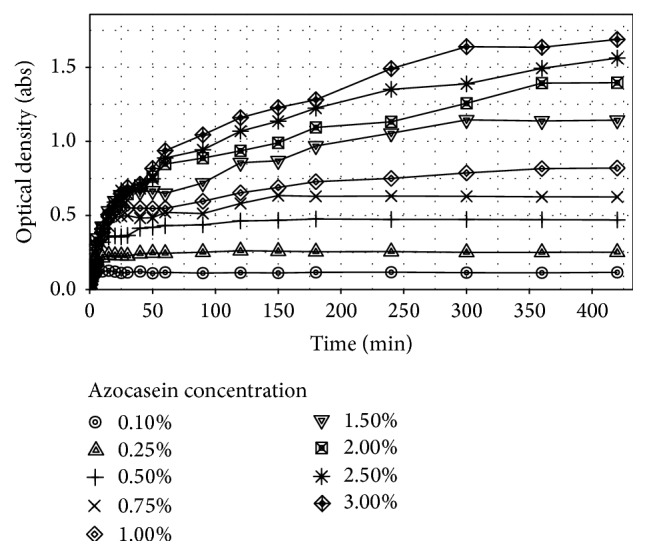
Azocasein digestion curve at 45°C and pH 9 using bromelain 1 mg/mL with substrate concentration from 0.1 to 3% (w/w).

Figure 2 shows the kinetics curves obtained for each concentration of azocasein substrate used. As expected, curves with lower substrate concentration were completely digested in a matter of a few minutes, while solution at 3%, 2.5%, and 2% seems to be closer to such point but the enzymatic reaction would still be in process.

**Figure 3:**
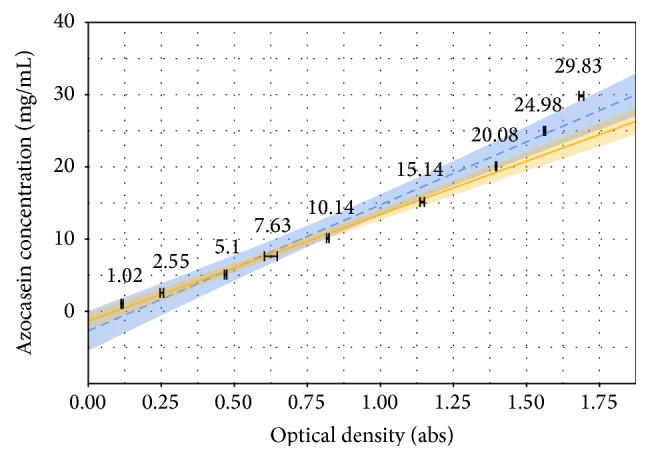
Calibration curves for azocasein concentration using 1~20 mg/mL (solid line, SL) and 1~30 mg/mL (dashed line, DL).

By plotting the azocasein concentration against its correspondent optical density for all curves at 420 min and using the assumption made by Charney and Tomarelli [6] we obtain a calibration curve which creates a relationship between these two variables (Figure 3).

The substrate concentration was converted easily from mass fraction to mg·mL^−1^ by taking in account the solvents specific mass and the volume retraction caused by the addition of ethanol.

The divergence between curves is mainly due the fact that reactions using substrate at 2.5% and 3.0% seem to have significant amounts of undigested substrate and thus the assumption becomes invalid. Therefore, the solid line (SL) curve represents the data series without these points. Results from statistical analysis for both curves are presented in Table 2.

As the presented data suggests, it is clear that removing the points related to unfinished reactions put the correlation in a confidence level allowing it to be used as a calibration curve. Consider

(2) $$\begin{array}{c} C_{AZO}\left(\;mg/mL\;\right)=-0.13561+1.47572\cdot Abs. \end{array}$$

The limits of detection and quantification were calculated using (1) and their results are presented below. Data was converted to mg·mL^−1^ using (2) and coefficients obtained for SL. Consider

(3) LoD3.3·σs=3.3·0.062951.47572=0.1407686Abs=0.072 mg/mLLoQ10·σs=10·0.062951.47572=0.4265714Abs=0.494 mg/mL.

One unit (U) of proteolytic activity was defined as the amount of enzyme capable of digesting 1 mg of substrate per minute, as given in the equation below:

(4) $$\begin{array}{c} A_{\left(U\right)}=\frac{C_{AZO}\cdot V_{Total}^{2}}{t\cdot V_{ENZ}}, \end{array}$$

where *C*_AZO_ is the concentration of azocasein obtained using (2); *V*_Total_ is the sum of volumes of TCA, substrate, and enzyme solution (*V*_ENZ_) used in the digestion and *t* is the digestion time (in minutes).

### 3.3. Stability Assays

**Figure 4:**
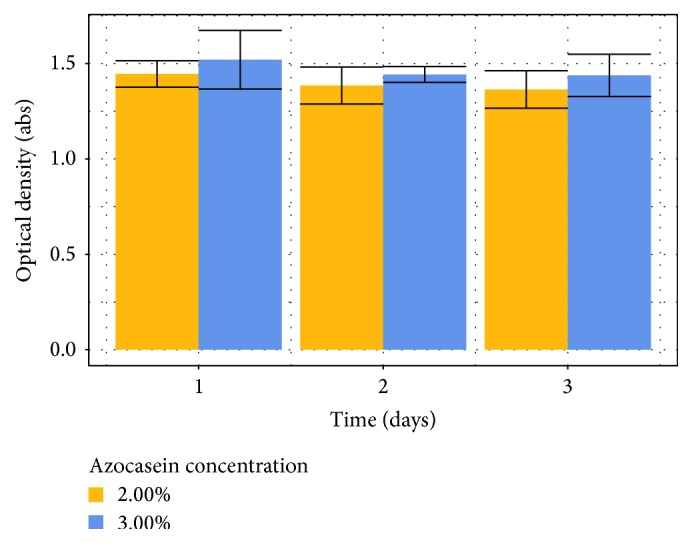
Short-term stability results for azocasein substrate.

Substrate's storage stability is another important feature to be evaluated in order to establish a protocol. Short-term stability is important to evaluate whether the substrate can be kept at room temperature during a daylong set of experiments (Figure 4).

Results of time = 0 are relative to a substrate solution right after it was prepared, while subsequent days showed results of each sample, taken from the same stock solution, left for 8 hours at room temperature prior to analysis. Results show a significant loss of substrate response in both concentrations (around 10%) when compared to the stock solution but that a similar variation is observed within the time interval studied.

Long-term stability is evaluated to check whether a solution can be stored and for how long, without been frozen.

**Figure 5:**
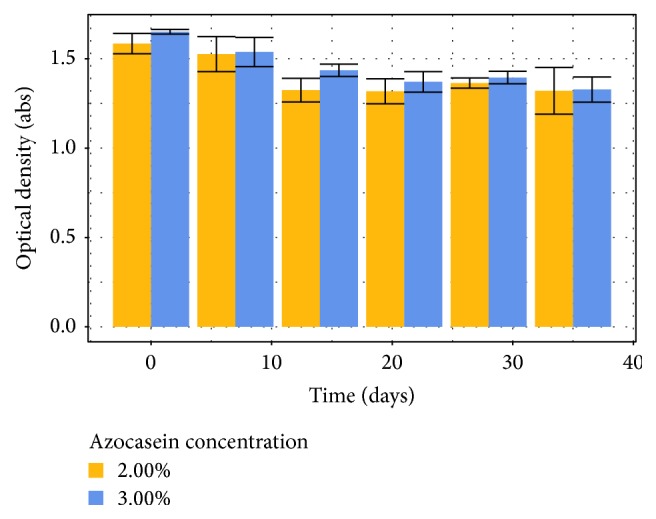
Long-term stability for azocasein substrate stored at 5°C.

While there was no observed formation of insoluble solids in the stock solution during storage, the response of substrate had a significant loss (around 17%) after 14 days but then it stabilized (Figure 5). This fact does not seem to create any interference in any step of the method but suggests that the substrate solution would offer a maximum response when used right after preparation. Further studies will be necessary to understand the phenomena involved in the decrease of response over time.

**Figure 6:**
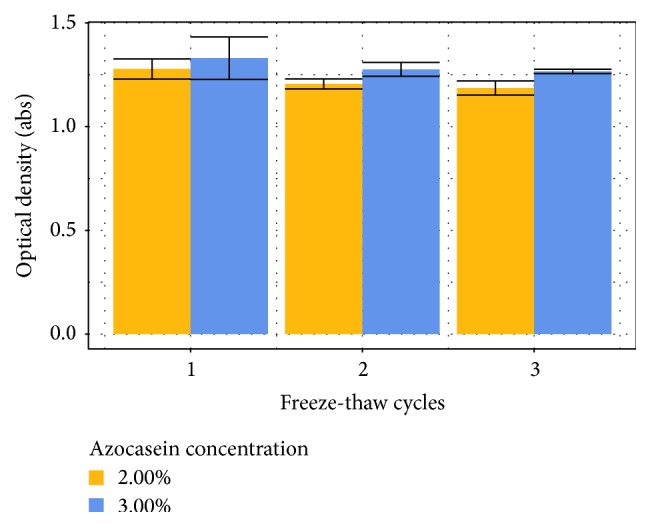
Substrate stability after freeze-thaw cycles.

The decrease in response for the substrate's digestion also occurred during freeze-thaw cycle (see Figure 6), which reinforces the hypothesis that it is not caused by microbial activity but somehow related to the substrate solubility. The observed errors were lower than the ones observed during long-term and short-term studies, which make it the most suitable option for storage at the moment.

## 4. Conclusion

The protocol described followed the main guidelines presented by ICH^*∗*^ and establishes a reliable procedure to analyse biological activity of proteolytic enzymes. Besides, the method uses a mass correlation between the substrate used and the optical density observed in the postdigestion sample. Although a simple and obvious idea, it offers a significant improvement, given that subjective definitions are commonly used in the literature. Besides, we ran a series of stability assays in order to evaluate the substrate and observed that a significant loss (10%–20%) occurred in all substrate samples, suggesting that substrate solution offers an enhanced response when prepared right after its use. As the understanding of the mechanism controlling the loss in substrate response was not part of this research, further experiments will be performed and analysed separately.

## Figures and Tables

**Table 1:** Rotational central composite design used to study and determine assay's optimum conditions shown in Figure 1(c).

	Factor	Temperature (°C)	pH
Levels	−1.414	37.93	6.17
	−1.000	40.00	7.00
	0.000	45.00	9.00
	1.000	50.00	11.00
	1.414	52.07	11.80

**Table 2:** Summary of statistical analysis results for both curves.

		Coefficients	Std. error	*t*-value	*R* ^2^
Solid line (SL)	Intercept	−0.13561	0.04493	3.018	0.9916
	Slope	1.47572	0.05533	26.673	

Dashed line (DL)	Intercept	−0.2700	0.1161	2.326	0.9687
	Slope	1.7441	0.1106	15.764	

## Nomenclature

ICH: International Conference on Harmonisation of Technical Requirements for Registration of Pharmaceuticals for Human Use.
